# Nonlinear association between triglyceride-glucose index and 28-day mortality in intensive care units: a multi-center retrospective cohort study

**DOI:** 10.3389/fendo.2025.1545478

**Published:** 2025-04-29

**Authors:** Bo Li, Xiaoan Yang, Jiang Hua Wang, Weidong Chen, Qi Wang, Lintao Zhong

**Affiliations:** ^1^ Department of Cardiology, the Sixth Affiliated Hospital, School of Medicine, South China University of Technology, Foshan, China; ^2^ Department of Infectious Diseases, the 3rd Affiliated Hospital, Sun Yat-Sen University, Guangzhou, China; ^3^ Nutrition of Cardiology, the Sixth Affiliated Hospital, School of Medicine, South China University of Technology, Foshan, China

**Keywords:** triglyceride-glucose index, insulin resistance, emergency department, intensive care unit, 28-day mortality, eICU database

## Abstract

**Background:**

The triglyceride-glucose (TyG) index, derived from the calculation of two biomarkers, fasting plasma glucose and triglyceride levels, is a reliable indicator of insulin resistance and has been demonstrated to be associated with the adverse clinical outcomes of patients in the intensive care unit (ICU). This study aims to investigate the relationship between the TyG index and the 28-day all-cause mortality of these patients during their ICU stay.

**Methods:**

This study employed a multicenter retrospective cohort design, analyzing data from 18,883 ICU patients in the eICU database. We calculated the TyG index for each patient and assessed its association with 28-day all-cause mortality. The Cox proportional hazards model was utilized for analysis, adjusting for various clinical and laboratory variables to control for confounding factors. We performed sensitivity analyses, subgroup analyses, and interaction analyses to evaluate the robustness of the results.

**Results:**

The study identified a significant positive correlation between the TyG index and 28-day all-cause mortality. Specifically, each one-unit increase in the TyG index corresponded to a 58% increase in mortality risk (HR=1.58, 95% CI: 1.25–2.00, P=0.0001). Additionally, the analysis revealed a non-linear threshold effect of the TyG index on mortality, with a cutoff point at 8.82; mortality was lower below this value and significantly increased above it. Sensitivity and subgroup analyses indicated robust findings, while E-value analysis suggested resilience against unmeasured confounding.

**Conclusion:**

This study establishes the TyG index as an independent predictor of 28-day all-cause mortality in critically ill patients, highlighting its potential value in clinical management and risk assessment. By recognizing the non-linear effect of the TyG index, clinicians can more effectively adjust treatment strategies to reduce mortality among high-risk patients.

## Introduction

The high mortality rate of patients in the Intensive Care Unit (ICU) poses a significant challenge to global healthcare systems and economies. (1, 2). Early identification of critically ill patients and understanding the predictive factors for ICU mortality are crucial for improving outcomes in this population. Currently, commonly used prognostic tools for assessing patient outcomes in the ICU include the Acute Physiology and Chronic Health Evaluation II (APACHE II), the Sequential Organ Failure Assessment (SOFA), and the Simplified Acute Physiology Score II (SAPS II) (3–5). However, these tools have notable limitations. These tools typically require extensive physiological data, which may be difficult to obtain in real-time, thus hindering urgent assessments. Additionally, both APACHE II and SOFA inadequately consider metabolic disturbances. Therefore, there is a growing demand for alternative or supplementary biomarkers.

Insulin resistance (IR) is a prevalent condition among critically ill patients and is considered a marker of systemic inflammatory response and metabolic dysregulation (6). Studies have shown that insulin resistance is closely related to various diseases such as cardiovascular diseases, cancer, and metabolic syndrome (7–9). Critically ill patients frequently exhibit decreased insulin sensitivity, which is closely associated with the severity of their illness (10, 11). However, insulin resistance in critically ill patients may fluctuate dynamically due to disease progression, treatment response, and nutritional status. As a result, short-term measurement of IR may not accurately reflect the overall clinical condition, thereby affecting the accuracy of mortality prediction (12). The TyG index, calculated by multiplying the fasting triglyceride level by the glucose concentration, has emerged as a novel biomarker for assessing insulin resistance and metabolic dysfunction (13, 14). The simplicity and accessibility of the TyG index have contributed to its widespread attention. Numerous studies have demonstrated the significant potential of the TyG index as a risk factor (15, 16). Additionally, research has confirmed that for patients with cardiovascular disease, the TyG index is significantly and independently associated with the risk of recurrent cardiovascular events, suggesting its great potential as a valuable prognostic predictor (17).

Several studies have confirmed the TyG index’s ability to predict mortality. A study conducted in the United States indicated that the TyG index can predict mortality in patients with cardiovascular comorbidities, diabetes, and prediabetes (18). Another study in China demonstrated that the TyG index serves as a predictor of all-cause mortality during long-term follow-up in older adults (19). Recent studies have highlighted a significant association between the TyG index and mortality in critically ill patients. Research has shown that a higher TyG index is generally linked to an increased risk of mortality in ICU patients (20). Furthermore, several studies have confirmed a significant relationship between the TyG index and mortality risk in patients with sepsis, cerebral infarction, coronary heart disease, and atrial fibrillation (21–23). Moreover, the TyG index also has predictive value for long-term all-cause mortality (24). However, previous studies have often involved small sample sizes, with most including fewer than 2,000 patients, making the results more susceptible to outliers and leading to instability in the findings. Additionally, the estimated margins of error were relatively large, which reduced the precision of the results. Moreover, most prior studies focused on specific diseases, which limits their ability to represent the prognostic relationship between the TyG index and all critically ill patients in the ICU, thereby restricting the generalizability of the findings.

Considering these gaps and limitations in the literature, our research aims to utilize the Philips eICU database to conduct a multicenter, large-sample retrospective cohort study to further investigate the relationship between the TyG index and the 28-day mortality of critically ill patients during their ICU stay. We aim to effectively address the limitations of previous studies and clarify the complex relationship between the TyG index and mortality, thereby enhancing the predictive capacity of ICU clinicians.

## Methods

### Study design

This study employs a retrospective cohort design to investigate the relationship between the TyG index and 28-day mortality in patients in the ICU. The data for this study were obtained from the eICU-CRD database, a multicenter ICU information repository known for its extensive representation and significant clinical relevance. This database includes clinical information from ICU patients admitted to over 200 hospitals in the United States from 2014 to 2015, providing treatment data and outcomes for more than 2 million patients. All collected data were anonymized and systematically archived through the Philips Healthcare eICU program, ensuring compliance with the Health Insurance Portability and Accountability Act (HIPAA) to enhance data security and protect patient privacy. To access the data, members of the research team completed the “Research with Data or Samples Only” course provided by the Collaborative Institutional Training Initiative (CITI). Consequently, this study was exempt from review by the Massachusetts Institute of Technology Institutional Review Board (IRB) (record number: 49995491) and from the requirement for informed consent. This research strictly adheres to the ethical guidelines outlined in the Declaration of Helsinki, confirming that all procedures involving human subjects comply with stringent ethical standards. We extracted and analyzed data regarding patients’ demographic information, clinical characteristics, laboratory test results, and treatment processes recorded in the eICU-CRD database.

### Selection of participants

**Figure 1:**
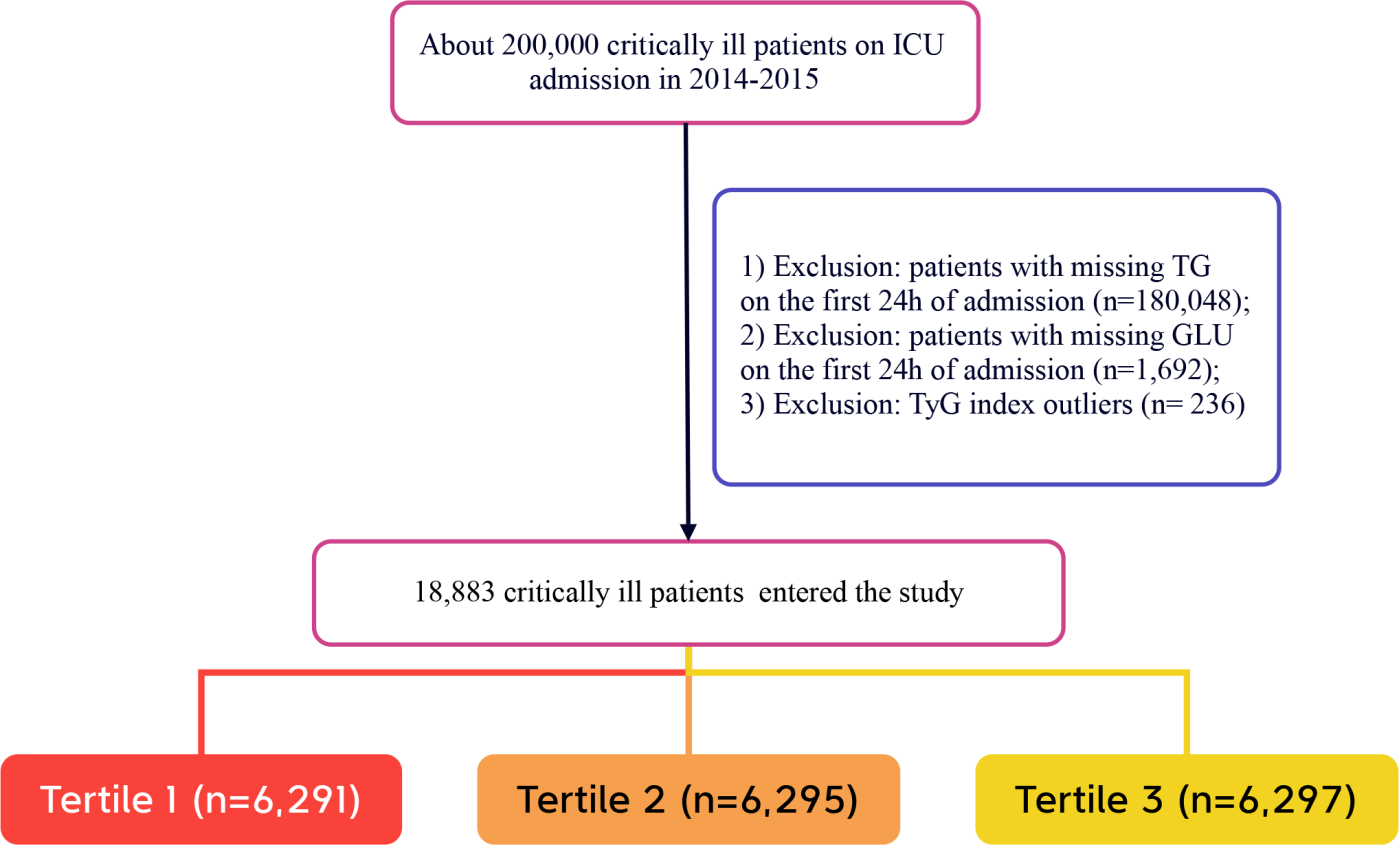
Flow chart of study population. ICU, intensive care unit.

The selection of participants in this study followed specific criteria. Inclusion criteria were: (1) adults aged 18 years and older who were in critical condition and admitted to the ICU. To ensure data integrity and the reliability of the study, the following exclusion criteria were established: (1) individuals without triglyceride test results on the day of admission; (2) individuals lacking glucose test results on the day of admission; (3) outliers in the TyG index, defined as individuals exceeding three standard deviations above the mean (25). After screening, a total of 18,883 participants were ultimately included in the study. These participants were further categorized into low TyG, medium TyG, and high TyG groups based on the tertiles of the TyG index recorded on the day of admission. The selection process for participants is illustrated in **Figure 1**, which clearly outlines the detailed steps of the screening process.

### Variable extraction

This study utilized data obtained from the eICU-CRD database, which included a variety of variables related to patients’ clinical conditions. The variables included demographic information—such as race, age, sex, height, weight, and body mass index (BMI)—as well as biochemical indicators, including total cholesterol, triglycerides, high-density lipoprotein cholesterol (HDL), low-density lipoprotein cholesterol (LDL), uric acid, creatinine, calcium, fasting blood glucose, and complete blood count. Comorbidities were identified using recorded International Classification of Diseases, 9th Revision (ICD-9) codes, which included common conditions such as diabetes mellitus (DM), chronic obstructive pulmonary disease (COPD), and acute myocardial infarction (AMI). Additionally, the severity of illness at admission was assessed using the SOFA score and the Glasgow Coma Scale (GCS) to quantify patients’ clinical status. All variables were collected within 24 hours of patient admission to ensure the data’s timeliness and relevance. If a variable was recorded multiple times during this period, the average value was calculated for analysis.

### Definition of TYG index and primary outcome

The TyG index is calculated based on initial fasting blood glucose and triglyceride levels using the following formula: TyG index = ln (fasting triglycerides (mg/dL) × fasting glucose (mg/dL)/2) (26, 27). The primary objective of this study is to assess the all-cause hospital mortality rate of patients during their 28-day stay in the ICU.

### Statistical analysis

In this study, we described the data. Continuous variables with a normal distribution were expressed as means and standard deviations, while those without a normal distribution were represented by medians and interquartile ranges. Categorical variables were presented according to their frequency and proportion, enhancing the clarity of the data distribution. We used the Student’s t-test and Pearson’s chi-square test, or Fisher’s exact test, to assess differences between variables, ensuring the results’ reliability and validity. We employed the Cox regression model to analyze the relationship between the TyG index and 28-day mortality, reporting hazard ratios (HR) and their 95% confidence intervals (CI) for clinical guidance. To control for potential confounding factors, we adjusted for several covariates, including sex, age, race, and BMI, enhancing the accuracy and rigor of the results. We utilized restricted cubic spline (RCS) models to explore the nonlinear relationship between the TyG index and ICU mortality risk. Additionally, we performed piecewise linear regression analysis to identify the threshold effect of the TyG index on ICU mortality and assessed model fit using likelihood ratio tests compared to simple linear regression. To ensure result validity, we conducted multiple analyses, including sensitivity, subgroup, and interaction analyses. We also calculated E-values to evaluate the potential impact of unmeasured confounders on the observed association between the TyG index and 28-day ICU mortality. The E-value calculation indicates the strength of unmeasured confounders required to nullify the observed effect (28, 29). We established the statistical significance level at a two-tailed α = 0.05. All statistical analyses were conducted using EmpowerStats (www.empowerstats.com, X&Y Solutions, Inc., Boston, MA) and R software version 3.6.1 (http://www.r-project.org).

## Results

### Baseline characteristics of the included participants

**Figure 2:**
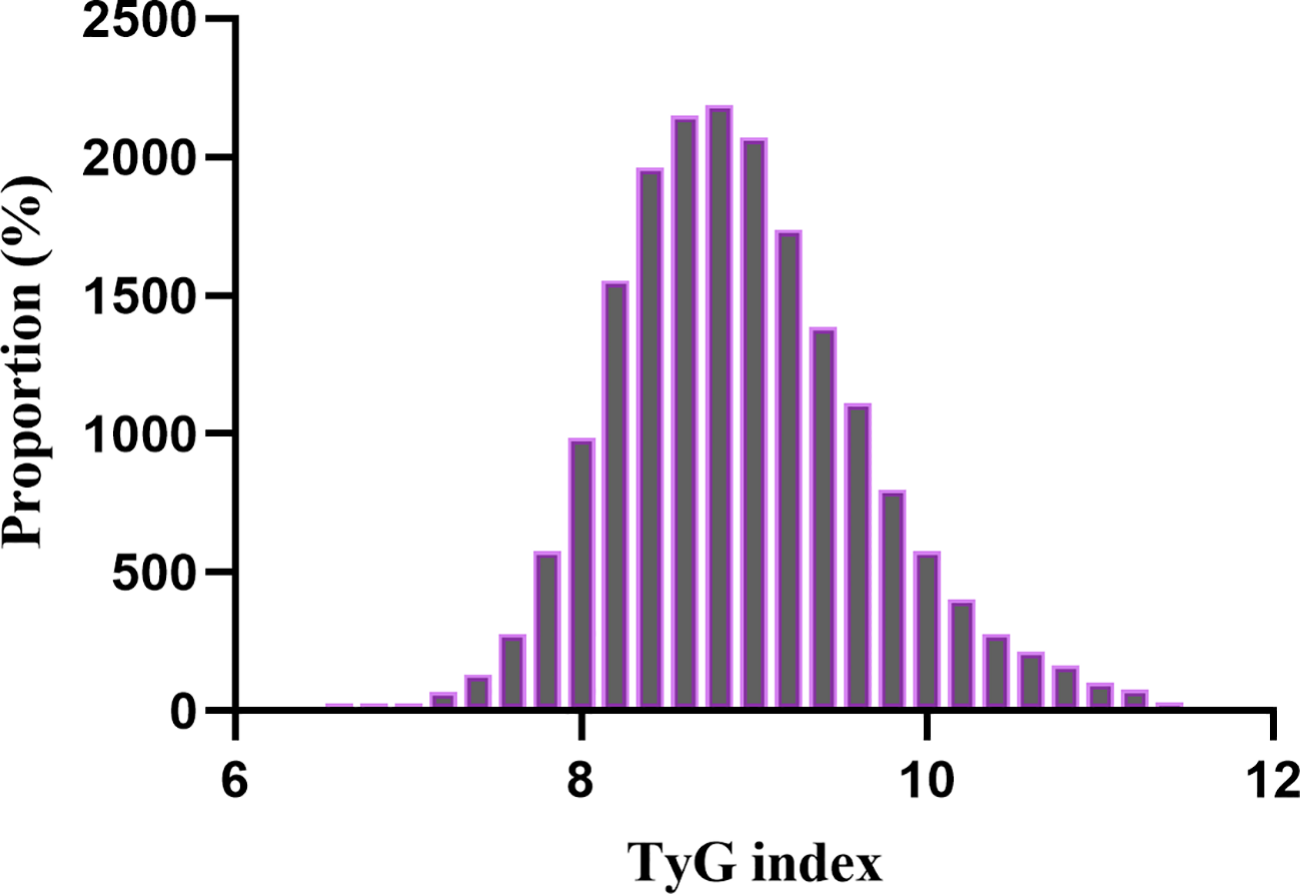
Distribution of TyG index. It presented a normal distribution, ranging from 6.54 to 11.39, with a mean of 8.92.

A total of 18,883 patients met the inclusion criteria in the eICU database (see **Figure 1**). The average age of the patients was 63.94 ± 15.35 years, with approximately 57.98% identifying as male. Among the ICU patients, 973 died within 28 days, representing 5.15% of the cohort. The baseline TyG index values ranged from 6.54 to 11.39, with a mean of 8.92 (see **Figure 2**). **Table 1** presents the baseline characteristics of participants categorized by tertiles of the TyG index. As the TyG index increased, notable trends were observed in various baseline parameters and clinical outcomes. Participants in the highest tertile were generally younger, had a higher BMI, and included a significantly greater proportion of male and white patients. Regarding biochemical markers, levels of blood glucose, blood urea nitrogen, and creatinine significantly increased with higher TyG index values, while HDL levels showed a decreasing trend. The incidence of comorbidities, including diabetes, sepsis, and acute myocardial infarction, significantly increased in higher TyG index tertiles. The 28-day ICU mortality rate showed significant differences across the TyG index tertiles. Compared to patients with lower TyG index values, those in higher TyG index tertiles had significantly longer ICU stays and higher mortality rates.

**Table 1:** The baseline characteristics of participants according to tertiles of TyG index.

Characteristics	T1(6.54-8.57)	T2(8.57-9.16)	T3(9.16-11.39)	P-value
Age(year)	66.59 ± 16.20	64.69 ± 14.78	60.54 ± 14.39	<0.001
BMI (kg/m^2^)	27.48 ± 7.38	29.65 ± 7.98	31.30 ± 8.22	<0.001
Gender				0.278
Male, n (%)	3591 (57.08%)	3673 (58.35%)	3668 (58.25%)	
Female, n (%)	2700 (42.92%)	2622 (41.65%)	2629 (41.75%)	
Ethnicity				<0.001
Caucasian, n (%)	4553 (72.37%)	4710 (74.82%)	4645 (73.77%)	
African-American, n (%)	951 (15.12%)	771 (12.25%)	714 (11.34%)	
Hispanic, n (%)	363 (5.77%)	363 (5.77%)	439 (6.97%)	
Asian, n (%)	227 (3.61%)	256 (4.07%)	269 (4.27%)	
Native American, n (%)	40 (0.64%)	26 (0.41%)	41 (0.65%)	
Unknown, n (%)	157 (2.50%)	169 (2.68%)	189 (3.00%)	
SOFA score, median (IQR)	2.00 (1.00-4.00)	1.00 (0.00-4.00)	2.00 (1.00-4.00)	<0.001
GCS score	13.26 ± 3.12	13.09 ± 3.36	12.66 ± 3.80	<0.001
BUN, median (IQR), (mmol/L)	16.0 (12.0-24.0)	17.0 (12.0-25.0)	19.0 (13.0-30.0)	<0.001
Serum calcium	8.49 ± 0.70	8.50 ± 0.71	8.48 ± 0.80	0.232
GLU, median (IQR), (mg/dl)	106.0 (92.0-124.0)	123.0 (104.5-150.0)	168.0 (127.0-233.0)	<0.001
HGB, (g/L)	11.87 ± 2.17	12.12 ± 2.30	12.26 ± 2.36	<0.001
PLT, (×10^9^/L)	205.75 ± 86.64	214.43 ± 86.32	219.92 ± 84.74	<0.001
TC, (mg/dl)	139.70 ± 41.07	152.79 ± 45.69	168.14 ± 52.67	<0.001
TG, median (IQR), (mg/dl)	68.0 (54.0-83.0)	112.0 (91.50-134.0)	185.0 (140.0-249.0)	<0.001
HDL, (mg/dl)	47.99 ± 17.65	41.14 ± 14.83	35.99 ± 13.26	<0.001
LDL, (mg/dl)	78.30 ± 34.22	89.64 ± 40.00	92.76 ± 44.33	<0.001
RBC, (×10/^12^L)	3.97 ± 0.71	4.07 ± 0.74	4.12 ± 0.77	<0.001
RDW, (%)	14.73 ± 2.19	14.65 ± 2.16	14.54 ± 2.03	<0.001
WBC, (×10^9^/L)	10.14 ± 6.20	11.28 ± 6.03	12.74 ± 9.97	<0.001
Complication				
Sepsis, n (%)	214 (3.40%)	208 (3.30%)	303 (4.81%)	<0.001
DM, n (%)	397 (6.31%)	566 (8.99%)	1033 (16.40%)	<0.001
Immunosuppression, n (%)	84 (1.34%)	113 (1.80%)	120 (1.91%)	0.031
Cirrhosis, n (%)	79 (1.26%)	56 (0.89%)	41 (0.65%)	0.002
COPD, n (%)	338 (5.37%)	344 (5.46%)	298 (4.73%)	0.131
CRF, n (%)	580 (9.22%)	520 (8.26%)	464 (7.37%)	<0.001
AMI, n (%)	982 (15.61%)	1304 (20.71%)	1340 (21.28%)	<0.001
28-day ICU mortality				<0.001
No, n (%)	6061 (96.34%)	5978 (94.96%)	5871 (93.23%)	
Yes, n (%)	230 (3.66%)	317 (5.04%)	426 (6.77%)	
ICU LOS, median (IQR), d	1.75 (1.00-3.08)	1.79 (1.01-3.42)	1.84 (1.02-3.82)	<0.001

Continuous variables are summarized as mean (SD) or median (trisection interval); categorical variables are presented as percentages (%). BMI, body mass index; COPD, chronic obstructive pulmonary disease; AMI, acute myocardial infarction; ICU, intensive care unit; LOS, length of stay; BUN, blood urea nitrogen; RBC, red blood cell; HGB, hemoglobin; PLT, platelets; SOFA, sequential organ failure assessment; GCS, glasgow coma scale; GLU, glucose; TC, total cholesterol; TG, triglycerides; HDL, high-density lipoprotein; RDW, red cell distribution width; WBC, white blood cells; CRF, chronic renal failure.

### Kaplan-Meier survival curve analysis of the TyG index and 28-day mortality

**Figure 3:**
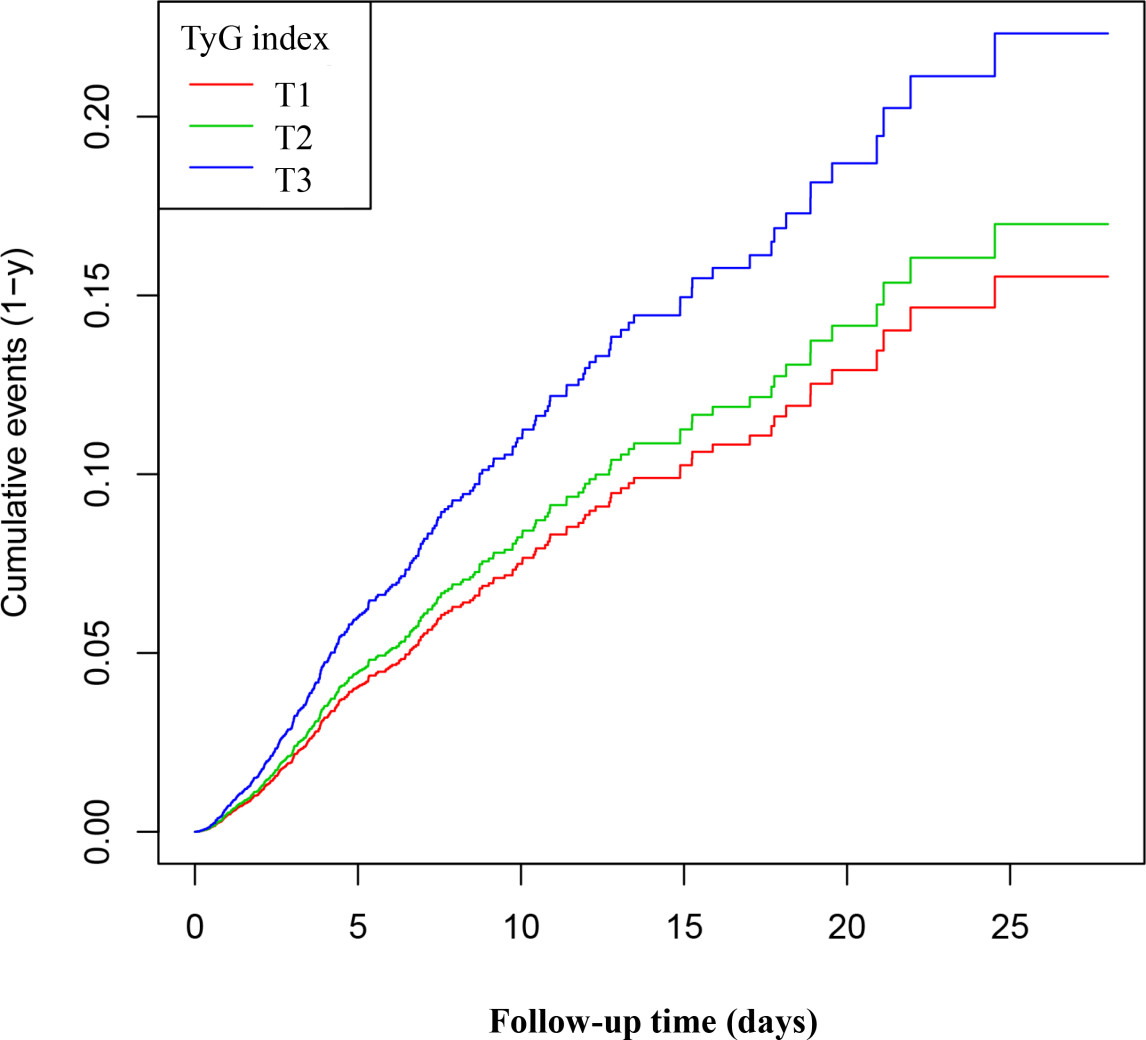
Kaplan–Meier curves for 28-day ICU. The probability of 28-day ICU mortality increased progressively with a rising TyG index, meaning that patients with the highest TyG index had a higher probability of ICU mortality within 28 days.

**Figure 3** displays the Kaplan-Meier survival curves for the TyG index and 28-day mortality. The results indicate that the 28-day mortality rate in the T1 group was significantly lower than in the T2 and T3 groups (P < 0.05). These findings suggest that a higher TyG index is associated with an increased risk of mortality within 28 days, highlighting its potential value in clinical prognostic assessment. Kaplan-Meier survival curves and the log-rank test indicated a significant difference in 28-day mortality among the three TyG index groups (P < 0.05). *Post hoc* pairwise comparisons with Bonferroni adjustment revealed that the 28-day mortality in the T1 group was significantly lower than that in the T2 and T3 groups (both comparisons P < 0.05), while the mortality in the T2 group was lower than that in the T3 group (P < 0.05).

### Univariate analysis

**Table 2** lists the factors influencing ICU mortality risk as identified through univariate Cox proportional hazards regression analysis. Age was identified as a significant predictor of ICU mortality risk, with older patients facing an increased risk. BMI demonstrated a slight protective effect, whereas sex did not significantly influence survival outcomes. Racial differences were observed, particularly with African American patients exhibiting a lower risk of hospital mortality. Clinical parameters, including the SOFA score, GCS score, and serum calcium levels, were associated with mortality risk. Higher SOFA scores were associated with an increased risk of both ICU and hospital mortality. Biochemical markers, including hemoglobin, red blood cell count, and the TyG index, were also significant predictors, suggesting their potential role in outcome prediction. Notably, complications such as sepsis, immunosuppression, and cirrhosis were linked to an increased risk of mortality.

**Table 2:** Factors influencing risk of ICU mortality analyzed by univariate Cox proportional hazards regression analysis.

Variable	Statistics	ICU mortality HR (95% CI)	P-value
Age (year)	63.94 ± 15.35	1.02 (1.01, 1.02)	<0.0001
BMI (kg/m2)	29.48 ± 8.02	0.99 (0.98, 1.00)	0.0068
Gender			
Female, n (%)	7951 (42.11%)	1.0	
Male, n (%)	10932 (57.89%)	1.00 (0.88, 1.13)	0.9744
Ethnicity			
Caucasian, n (%)	13908 (73.65%)	1.0	
African-American, n (%)	2436 (12.90%)	0.86 (0.72, 1.04)	0.1146
Hispanic, n (%)	1165 (6.17%)	0.98 (0.75, 1.28)	0.8601
Asian, n (%)	752 (3.98%)	0.84 (0.59, 1.17)	0.2979
Native American, n (%)	107 (0.57%)	1.24 (0.56, 2.78)	0.5942
Unknown, n (%)	515 (2.73%)	1.02 (0.71, 1.48)	0.8998
SOFA score, median (IQR)	2.00 (1.00-4.00)	0.85 (0.84, 0.86)	<0.0001
GCS score	13.00 ± 3.44	0.85 (0.84, 0.86)	<0.0001
BUN, median (IQR), (mmol/L)	17.00 (12.00-26.00)	1.01 (1.01, 1.01)	<0.0001
Serum calcium	8.49 ± 0.74	0.82 (0.76, 0.89)	<0.0001
GLU, median (IQR), (mg/dl)	125.0 (103.0-164.0)	1.00 (1.00, 1.00)	<0.0001
HGB, (g/L)	12.08 ± 2.29	0.97 (0.94, 0.99)	0.0086
PLT, (×109/L)	213.36 ± 86.10	1.00 (1.00, 1.00)	0.2911
TC, (mg/dl)	153.22 ± 48.00	1.00 (0.99, 1.00)	<0.0001
TG, median (IQR), (mg/dl)	108.0 (75.0-159.0)	1.00 (1.00, 1.00)	0.1060
LDL, (mg/dl)	86.64 ± 40.03	1.00 (0.99, 1.00)	0.0020
HDL, (mg/dl)	41.86 ± 16.18	0.99 (0.99, 1.00)	0.0009
RBC. (×10/12L)	4.05 ± 0.74	0.91 (0.84, 0.99)	0.0213
RDW (%)	14.64 ± 2.13	1.09 (1.07, 1.12)	<0.0001
WBC, (×109/L)	11.38 ± 7.69	1.01 (1.00, 1.01) <	<0.0001
TyG index	8.92 ± 0.73	1.21 (1.11, 1.31)	<0.0001
Complication			
Sepsis, n (%)	725 (3.84%)	1.40 (1.10, 1.77)	0.0055
DM, n (%)	1996 (10.57%)	0.83 (0.67, 1.03)	0.0865
Immunosuppression, n (%)	317 (1.68%)	1.82 (1.31, 2.53)	0.0004
Cirrhosis, n (%)	176 (0.93%)	1.83 (1.22, 2.74)	0.0036
COPD, n (%)	980 (5.19%)	0.81 (0.62, 1.07)	0.1332
CRF, n (%)	1564 (8.28%)	1.05 (0.86, 1.28)	0.6496
AMI, n (%)	3626 (19.20%)	1.00 (0.83, 1.20)	0.9898

CI, confidence interval; HR, hazard ratios.

### Multivariable analysis using Cox proportional hazards regression model

We utilized three Cox proportional hazards regression models to evaluate the impact of the TyG index on 28-day all-cause mortality in ICU patients. In the initial unadjusted model (Model I), each 1-unit increase in the TyG index was linked to a 21% increase in the likelihood of all-cause mortality, corresponding to a hazard ratio (HR) of 1.21 (95% CI: 1.11–1.31, P < 0.0001). In the partially adjusted model (Model II), which accounted for sex, age, and race, each 1-unit increase in the TyG index was associated with a 29% increase in mortality risk, resulting in an HR of 1.29 (95% CI: 1.19–1.41, P < 0.0001). The fully adjusted model (Model III) showed that each 1-unit increase in the TyG index was associated with a 58% increase in mortality risk, resulting in an HR of 1.58 (95% CI: 1.25–2.00, P = 0.0001). Consistent confidence intervals further reinforce the strong association between the TyG index and 28-day mortality in ICU patients, as demonstrated in **Table 3**. Additionally, we transformed the TyG index into categorical variables and reintroduced these variables into the models. The multivariable adjusted model revealed that, compared to participants in the first group (T1), those in the second and third groups (T2 and T3) had HRs of 1.10 and 1.50, respectively. This indicates that the likelihood of all-cause mortality for participants in the T2 and T3 groups increased by 10% and 50%, respectively (see **Table 3**, Model III).

**Table 3:** Relationship between TyG index and 28-day mortality in different models.

Exposure	Model I HR (95% CI) P-value	Model II HR (95% CI) P-value	Model III HR (95% CI) P-value
TyG index as continuous	1.21 (1.11, 1.31) <0.0001	1.29 (1.19, 1.41) <0.0001	1.58 (1.25, 2.00) 0.0001
TyG index tertiles			
T1	Ref	Ref	Ref
T2	1.24 (1.04, 1.47) 0.0142	1.28 (1.08, 1.51) 0.0048	1.10 (0.83, 1.47) 0.5030
T3	1.49 (1.27, 1.75) <0.0001	1.64 (1.39, 1.93) <0.0001	1.50 (1.06, 2.12) 0.0229
P for trend	<0.0001	<0.0001	0.0220

Model I: we did not account for additional variables.

Model II: we adjusted gender, age, ethnicity.

Model III: we adjusted gender, age, ethnicity, BMI, SOFA score, GCS score, serum calcium, BNU, HBG, TC, LDL, HDL, RBC, RDW, WBC, PLT, sepsis, immunosuppression and cirrhosis.

HR, Hazard Ratios; CI, confidence; Ref, reference.

### The results of sensitivity analysis

**Table 4** presents sensitivity analyses that examine the association between the TyG index and 28-day all-cause mortality in ICU patients. In Model I, the sensitivity analysis focused on patients younger than 60 years (N = 7,848). After adjusting for confounding variables, the TyG index was associated with an increased risk of 28-day all-cause mortality, resulting in a HR of 1.89 (95% CI: 1.13–3.14, P = 0.0147). Model II examined patients with a BMI < 25 kg/m² (N = 5,360). In this model, the TyG index was significantly associated with an increased risk of 28-day all-cause mortality, yielding an HR of 1.64 (95% CI: 1.05–2.54, P = 0.0282). In Model III, a generalized additive model (GAM) that incorporated smooth terms for multiple variables showed an HR of 1.58 (95% CI: 1.23–2.02, P = 0.0003). These extensive sensitivity analyses highlight the robustness of our findings. Additionally, we calculated E-values to evaluate the sensitivity to unmeasured confounding factors.

**Table 4:** Relationship between TyG index and 28-day ICU mortality in different sensitivity analyses.

Exposure	Model I HR (95% CI) P-value	Model II HR (95% CI) P-value	Model III HR (95% CI) P-value
TyG index as continuous	1.89 (1.13, 3.14) 0.0147	1.64 (1.05, 2.54) 0.0282	1.58 (1.23, 2.02) 0.0003
TyG index tertiles			
T1	Ref	Ref	Ref
T2	1.24 (0.63, 2.42) 0.5325	1.43 (0.86, 2.37) 0.1682	1.15 (0.86, 1.55) 0.3412
T3	2.16 (0.96, 4.84) 0.0626	1.94 (0.99, 3.79) 0.0524	1.49 (1.04, 2.14) 0.0304
P for trend	0.0590	0.0509	0.0301

Model I was a sensitivity analysis performed patients’ age<60 years (N= 7048). We adjusted gender, age, ethnicity, BMI, SOFA score, GCS score, serum calcium, BNU, HBG, TC, LDL, HDL, RBC, RDW, WBC, PLT, sepsis, immunosuppression and cirrhosis.

Model II was a sensitivity analysis performed patients’ BMI<25 kg/m2 (N= 5360). We adjusted gender, age, ethnicity, BMI, SOFA score, BUN, serum calcium, TC, HDL, RBC, HBG, WBC, RDW, hepatic failure, immunosuppression and cirrhosis.

Model III was adjusted gender, age (smooth), ethnicity, BMI (smooth), SOFA score (smooth), BUN (smooth), serum calcium (smooth), TC (smooth), HDL (smooth), RBC (smooth), HBG (smooth), WBC (smooth), RDW (smooth), hepatic failure, immunosuppression and cirrhosis.

### Identification of nonlinear relationship

**Figure 4:**
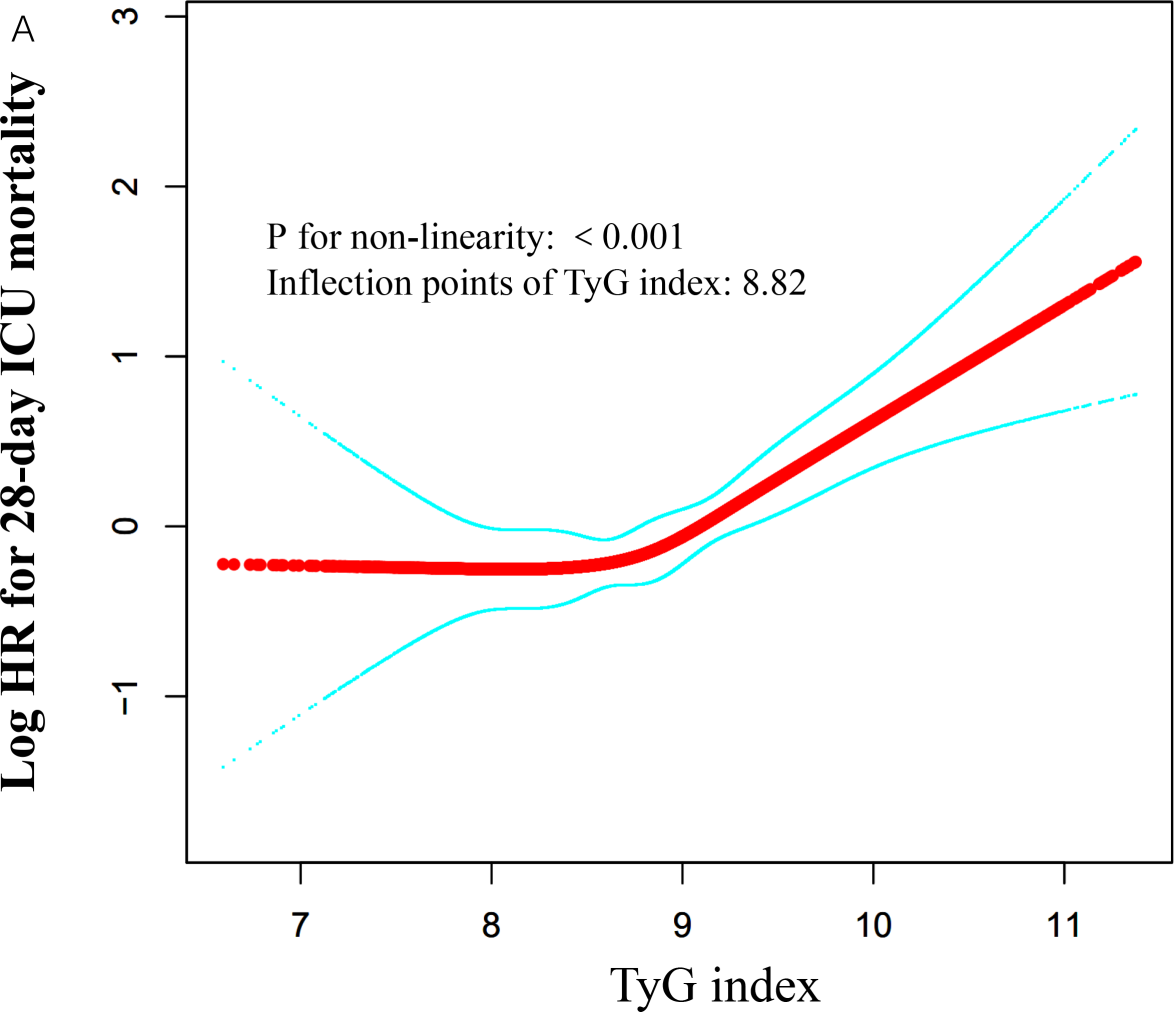
Associations between TyG index and 28-day mortality in critically ill patients in ICU. A threshold, nonlinear association between TyG index and 28-day mortality was identified using a GAM. The solid red line represents the smooth curve fitted between the variables. The blue bands represent the 95% CI from the fitted model. The analysis was adjusted for gender, age, ethnicity, BMI, SOFA score, BUN, serum calcium, TC, HDL, RBC, HBG, WBC, RDW, hepatic failure, immunosuppression and cirrhosis.

We employed a generalized additive model to identify a nonlinear threshold effect between the TyG index and 28-day all-cause mortality in the ICU (see **Figure 4**). We compared a linear regression model with a piecewise linear regression model. The likelihood ratio test results indicated that the relationship between the TyG index and 28-day all-cause mortality in the ICU was better fitted to the piecewise linear regression model than to the linear regression model (see **Table 5**), with a likelihood ratio P-value of <0.05. Specifically, when the TyG index is less than 8.82, a 1-unit increase does not lead to an increase in 28-day ICU all-cause mortality (adjusted HR 1.07, 95% CI: 0.73–1.56, P = 0.7324). However, when the TyG index is ≥ 8.84, a 1-unit increase corresponds to a 99% increase in 28-day mortality among ICU patients (adjusted HR 1.99, 95% CI: 1.48–2.66, P < 0.001).

**Table 5:** Threshold effect analysis of the TyG index and 28-day mortality.

Models	HR (95% CI)	P value
Model I		
One line effect	1.58 (1.25, 2.00)	0.0001
Model II		
Turning point (K)	8.82	
TyG < K	1.07 (0.73, 1.56)	0.7324
TyG ≥ K	1.99 (1.48, 2.66)	<0.0001
P value for LRT test*	0.015	

Data were presented as OR (95% CI) P value; Model I, linear analysis; Model II, non-linear analysis. Adjusted for age, ethnicity, AF; BMI; AMI; stroke; cancer; APACHE-IV score; BUN; Hb; serum calcium; mechanical ventilation. CI confidence interval, OR odds ratio. LRT logarithm likelihood ratio test. *P < 0.05 indicates that model II is significantly different from Model I.

### Subgroup analyses

We also investigated additional risk factors that may influence the relationship between the TyG index and 28-day ICU mortality risk. We selected age, sex, SOFA score, discharge time, and histories of DM, AMI, COPD, and CHF as stratification factors. Subsequently, we examined the trends in effect sizes for these factors (see **Table 6**). Our analysis revealed that age, sex, SOFA score, discharge time, and histories of diabetes, AMI, COPD, and CHF did not significantly impact the association between the TyG index and 28-day ICU mortality risk (all interaction P-values were greater than 0.05).

**Table 6:** Effect size of TyG index on 28-day ICU mortality in prespecified and exploratory subgroups.

Variable	Number	HR (95% CI) P	P for interaction
Age, yeas, Tertile			0.6839
<65	9265	1.51 (1.11, 2.05) 0.0079	
≥65	9618	1.61 (1.24, 2.08) 0.0003	
BMI (kg/m 2), Tertile			0.7174
T1 (≤25.55)	5999	1.68 (1.26, 2.26) 0.0005	
T2 (25.56-31.08)	5999	1.53 (1.11, 2.10) 0.0089	
T3 (≥31.08)	6000	1.46 (1.04, 2.03) 0.0276	
SOFA score			0.6845
T1 (0)	4557	2.32 (1.02, 5.27) 0.0439	
T2 (1-2)	7058	1.54 (0.95, 2.51) 0.0820	
T3 (≥3)	7265	1.67 (1.31, 2.13) <0.0001	
Gender			0.0574
Male, n	10932	1.44 (1.18, 1.76) 0.0004	
Female, n	7951	1.78 (1.44, 2.21) <0.0001	
Hospital discharge year			0.8192
2014, n	9143	1.56 (1.18, 2.06) 0.0018	
2015, n	9740	1.61 (1.22, 2.13) 0.0007	
COPD			0.5645
No, n	17903	1.60 (1.26, 2.03) 0.0001	
Yes, n	980	1.32 (0.69, 2.53) 0.3977	
CHF			0.8473
No, n	17319	1.57 (1.23, 2.00) 0.0003	
Yes, n	1564	1.64 (1.06, 2.54) 0.0272	
AMI			0.5668
No, n	15257	1.54 (1.20, 1.98) 0.0007	
Yes, n	3626	1.71 (1.19, 2.48) 0.0042	
DM			0.5599
No, n	16887	1.69 (1.32, 2.16) <0.0001	
Yes, n	1996	1.49 (0.97, 2.29) 0.0718	

Above model adjusted for gender, age, ethnicity, BMI, SOFA score, GCS score, serum calcium, BNU, HBG, TC, LDL, HDL, RBC, RDW, WBC, PLT, sepsis, immunosuppression and cirrhosis. In each case, the model is not adjusted for the stratification variable when the stratification variable was a categorical variable.

## Discussion

This study establishes the association between the TyG index and 28-day all-cause mortality in critically ill ICU patients. We conducted a multicenter retrospective cohort study involving 18,883 patients. Our findings reveal a significant positive correlation between the TyG index and 28-day mortality, which remained significant after adjusting for various clinical and laboratory variables. We also identified a nonlinear threshold effect, with a threshold point at 8.82; below this point, the mortality rate was low, while above it, the rate increased rapidly. This suggests that the TyG index may be a crucial prognostic indicator for critically ill patients, potentially influencing clinical management and risk assessment strategies.

It is well known that the ICU has the highest mortality rates in most hospitals due to various factors. First, ICU patients often present with severe conditions and multiple comorbidities, such as hypertension, diabetes, and malignancies, which significantly increase their risk of death (30–32). Additionally, common complications among ICU patients, such as sepsis, heart failure, and stroke (33–35), also significantly contribute to high mortality rates. Annually, approximately 4 million ICU admissions occur in the United States alone, with average mortality rates ranging from 8% to 19%. ICU mortality rates are lower in developed regions, including North America (9.3%), Oceania (10.3%), and Europe (18.7%). In developed Asia, the ICU mortality rate is 13.7%. In contrast, it is higher in South America (21.7%) and the Middle East (26.2%), and may reach as high as 53.6% in underdeveloped regions of Africa (36). In our study, there were 973 deaths among ICU patients within 28 days, representing 5.15% of the total. This may be associated with shorter hospital stays and fewer complications and comorbidities. One study suggested that the length of ICU stay affects mortality, with longer stays correlating with higher risks of death. The average length of stay for all patients in our study was 3.16 days, which may explain the observed lower mortality rate. Additionally, our study did not capture mortality rates outside the ICU, which may contribute to the lower mortality rate compared to the U.S. average. Overall, ICU mortality rates remain high, particularly in underdeveloped regions, highlighting the critical need for a cost-effective, rapid, and effective predictive indicator to reduce mortality in critically ill patients.

IR is a complex pathophysiological process marked by impaired glucose handling despite normal or elevated serum insulin levels (37). IR significantly contributes to several metabolic diseases, including type 2 diabetes, cardiovascular diseases (CVD), and obesity (38–40). Additionally, IR is closely associated with aging. As individuals age, they often experience insufficient insulin secretion, decreased glucose tolerance, and heightened IR due to muscle atrophy, obesity, and osteoporosis (41). Recent studies suggest that IR is not merely a consequence of a specific disease but rather a universal response to critical illness (6). The TyG index has recently been recognized as a simple and reliable surrogate marker for IR. Previous studies confirm that the TyG index highly correlates with the hyperinsulinemic-euglycemic clamp technique and shows greater assessment value than the traditional homeostasis model assessment of IR (HOMA-IR) (42, 43).

The TyG index, as a surrogate marker of insulin resistance, is closely associated with various cardiovascular diseases. Studies have shown that an elevated TyG index is positively correlated with the severity of obstructive sleep apnea (OSA) (44), potentially exacerbating cardiovascular risk through systemic inflammation and oxidative stress. In patients with atrial fibrillation (45), a high TyG index is linked to atrial remodeling and electrophysiological disturbances, increasing the incidence and recurrence of arrhythmias. Additionally, the TyG index is significantly associated with the severity and poor prognosis of heart failure (HF) (46), likely due to aggravated myocardial metabolic dysfunction and fibrosis. In peripheral arterial disease (47), a higher TyG index correlates with the progression of atherosclerosis and an increased risk of limb ischemia. The potential mechanisms underlying the association between the TyG index and high cardiovascular risk include metabolic disorders induced by insulin resistance, chronic inflammation, endothelial dysfunction, and enhanced oxidative stress, all of which collectively contribute to the development and progression of cardiovascular diseases.

Recent studies indicate a strong association between the TyG index and critically ill patients. Cai et al. (17) demonstrated that among 733 stroke patients, the TyG index was significantly associated with ICU mortality, with an elevated TyG index correlating with a higher mortality risk (HR of 1.653). Zhang et al. (23) found that in a retrospective cohort study of 1,618 patients with severe coronary artery disease, the TyG index was significantly associated with ICU mortality (HR of 1.50). Zheng et al. (22) indicated that in a cross-sectional study of 1,257 sepsis patients, a higher TyG index was associated with increased in-hospital mortality among critically ill patients. These findings necessitate larger prospective cohort studies for validation. However, these studies typically focused on specific diseases with smaller sample sizes, possibly limiting their applicability to the broader ICU patient population. Thus, the relationship between the TyG index and mortality risk in ICU patients warrants further investigation. To our knowledge, only one study (20) has evaluated the relationship between the TyG index and all-cause mortality in critically ill patients, suggesting that the TyG index may be a valuable tool for identifying high-risk ICU patients. However, that study had a sample size of only 3,026. Although it adjusted for many covariates, it did not perform E-value assessments for unmeasured confounders or conduct nonlinear testing. Therefore, a large cohort study is needed to further validate the association between the TyG index and mortality risk in critically ill patients. Our study found that the TyG index is an independent predictor of ICU mortality, consistent with previous literature (20). We screened 18,883 critically ill patients from ICUs across more than 200 hospitals in the United States, significantly exceeding previous study sample sizes. This enhanced the power to test hypotheses and facilitated the discovery of true effects or associations. Another significant finding is the identification of a nonlinear threshold effect between the TyG index and 28-day all-cause mortality, with a threshold point at 8.82. Below this threshold, the mortality rate remains low. Above it, ICU patient mortality increases rapidly. This finding has significant clinical implications. By recognizing this nonlinear effect, healthcare providers can modify treatment strategies based on changes in the TyG index. For instance, when the TyG index approaches or exceeds the threshold, enhanced monitoring and intervention may be necessary to reduce the risk of complications and mortality.

In subgroup analyses, Liao et al. (20) found the predictive value of the TyG index to be more pronounced in non-diabetic patients [HR (95% CI): Non-diabetic 2.83 (1.95–4.12) vs. Diabetic 0.81 (0.34–1.92), interaction P-value = 0.013]. They also noted that patients with higher TyG indices tended to be younger, and the association between the TyG index and all-cause mortality was more evident in this group, contradicting traditional views. Accordingly, we conducted a similar subgroup analysis, and our results revealed some differences compared to their conclusions. Our analysis also indicated that the association between the TyG index and all-cause mortality was somewhat more pronounced in non-diabetic patients; however, no interaction was detected, suggesting that both findings are fundamentally consistent. Additionally, our study found that patients with higher TyG indices were younger (see **Table 1**). In older patients, the association between the TyG index and all-cause mortality was slightly more pronounced; however, no interaction was detected (see **Table 6**). The discrepancy between Liao et al.’s findings and traditional views may primarily result from an insufficient sample size and a failure to adjust for necessary variables. The conclusions of our study are more reliable and stable. To account for unpredictable covariates, we assessed E-values, effectively ruling out the influence of most unmeasured variables.

The mechanisms underlying the association between the TyG index and 28-day all-cause mortality in ICU patients remain unclear. We reviewed a significant body of literature suggesting that the IR indicated by the TyG index is associated with the severity of illness. IR can cause disturbances in glucose and lipid metabolism, leading to increased systemic inflammation and oxidative stress, which may elevate mortality risk in ICU patients (20). Studies indicate that the TyG index is closely linked to the prognosis of CVD (48). A high TyG index may increase all-cause mortality in ICU patients by promoting atherosclerosis and impairing endothelial function (49, 50). The TyG index is associated with inflammation, which can induce metabolic imbalances in glucose and lipids, resulting in chronic hyperglycemia and dyslipidemia (14, 51). These conditions significantly contribute to the deterioration of ICU patients’ health.

Our findings suggest that the TyG index is associated with mortality in ICU patients. The TyG index is more readily obtainable compared to other indices, making it advantageous for clinicians to utilize in rapidly assessing patient conditions. The nonlinear relationship between the TyG index and ICU mortality, along with the identification of a cutoff value, enables clinicians to quickly distinguish between low-risk and high-risk patients and implement necessary clinical interventions for high-risk patients in a timely manner. Our analysis also indicates that the association between the TyG index and all-cause mortality is more pronounced in non-diabetic patients, which may be related to clinicians being more likely to overlook insulin resistance in non-diabetic individuals. The study results remind clinicians to pay equal attention to insulin resistance in non-diabetic patients and address it promptly. Additionally, our research found that patients with higher TyG indices tend to be younger, suggesting that clinicians should also prioritize insulin resistance in younger ICU patients.

## Strengths and limitations

Our study possesses several key strengths. First, we confirmed a positive correlation between the TyG index and 28-day all-cause mortality in ICU patients and identified a nonlinear threshold effect. This specific threshold (8.82) allows clinicians to conduct personalized assessments based on patients’ TyG indices. When the index exceeds this threshold, the mortality risk significantly increases, necessitating enhanced monitoring and treatment for these patients. Second, our study, which included a large sample of 18,883 patients, employed a multicenter retrospective cohort design, thereby improving the representativeness and applicability of the findings compared to smaller sample or case-control studies. Third, we performed comprehensive sensitivity analyses to validate the reliability of the TyG index as a predictor of mortality risk, further enhancing the credibility of our results. Fourth, our study included subgroup analyses and interaction assessments to deepen our understanding of the performance of the TyG index across various patient populations. Finally, our analysis considered multiple potential confounding factors, including SOFA scores, GCS scores, RDW, white blood cell counts, platelet counts, sepsis, immunosuppression, and cirrhosis. We also employed E-values to assess sensitivity to unmeasured confounding factors. Many prior studies may have produced biased results due to insufficient adjustments for these factors.

However, this study has several limitations. First, although the data were collected from over 200 hospitals in the United States involving 18,883 patients, the results may not be generalizable to ICU patients in other countries or regions, limiting their broader applicability. Second, as a retrospective study, it may be subject to selection and information biases, which could influence the interpretation of results and the reliability of conclusions, particularly during patient selection and data collection. Third, despite controlling for various confounding factors, unidentified variables may still exist. We conducted E-value sensitivity analyses to evaluate the potential impact of unmeasured confounders, with results indicating that these factors are unlikely to fully account for the treatment effects. Fourth, this study did not explore the biological mechanisms linking the TyG index to mortality in critically ill patients, limiting our understanding of its role as a prognostic indicator. Future research should focus on these physiological mechanisms. Finally, due to database limitations, we could not verify whether all glucose measurements were obtained from fasting samples. Although our study results are representative, further validation of their applicability in independent cohorts is necessary.

## Conclusion

This study reveals a significant positive correlation between the TyG index and 28-day all-cause mortality, even after adjusting for various clinical and laboratory variables. We also identified a nonlinear threshold effect between the TyG index and 28-day all-cause mortality, with a threshold set at 8.82. Below this threshold, the mortality rate remains low; however, above it, the mortality rate among patients in the ICU increases sharply. These findings suggest that the TyG index may serve as a crucial prognostic indicator for critically ill patients and could influence clinical management and risk assessment strategies.

## Data Availability

Publicly available datasets were analyzed in this study. This data can be found here: https://eicu-crd.mit.edu/.
